# The Mechanism of Antifungal Action of Essential Oil from Dill (*Anethum graveolens* L.) on *Aspergillus flavus*

**DOI:** 10.1371/journal.pone.0030147

**Published:** 2012-01-17

**Authors:** Jun Tian, Xiaoquan Ban, Hong Zeng, Jingsheng He, Yuxin Chen, Youwei Wang

**Affiliations:** Key Laboratory of Combinatorial Biosynthesis and Drug Discovery, Ministry of Education, School of Pharmaceutical Sciences, Institute of Traditional Chinese Medicine & Natural Products, Wuhan University, Wuhan, P. R. China; Newcastle University, United Kingdom

## Abstract

The essential oil extracted from the seeds of dill (*Anethum graveolens* L.) was demonstrated in this study as a potential source of an eco-friendly antifungal agent. To elucidate the mechanism of the antifungal action further, the effect of the essential oil on the plasma membrane and mitochondria of *Aspergillus flavus* was investigated. The lesion in the plasma membrane was detected through flow cytometry and further verified through the inhibition of ergosterol synthesis. The essential oil caused morphological changes in the cells of *A. flavus* and a reduction in the ergosterol quantity. Moreover, mitochondrial membrane potential (MMP), acidification of external medium, and mitochondrial ATPase and dehydrogenase activities were detected. The reactive oxygen species (ROS) accumulation was also examined through fluorometric assay. Exposure to dill oil resulted in an elevation of MMP, and in the suppression of the glucose-induced decrease in external pH at 4 µl/ml. Decreased ATPase and dehydrogenase activities in *A. flavus* cells were also observed in a dose-dependent manner. The above dysfunctions of the mitochondria caused ROS accumulation in *A. flavus*. A reduction in cell viability was prevented through the addition of L-cysteine, which indicates that ROS is an important mediator of the antifungal action of dill oil. In summary, the antifungal activity of dill oil results from its ability to disrupt the permeability barrier of the plasma membrane and from the mitochondrial dysfunction-induced ROS accumulation in *A. flavus*.

## Introduction

Members of the genus *Aspergillus* are ubiquitous filamentous fungus found anywhere on earth. To date, over 185 *Aspergillus* species have been identified, 20 of which have been reported to cause harmful infections in humans, animals, and plants. Among the *Aspergillus* species, the *Aspergillus flavus* may be the most infamous because it causes direct infections and systematic diseases in humans [1]. *A. flavus* is second only to *A. fumigatus*, the leading cause of human invasive aspergillosis [2]. Although invasive aspergillosis is rare in immunocompetent people, it contributes to the morbidity and mortality rate in immunosuppressed patients [3]. In addition, *A. flavus* is frequently the main cause of food contamination and the key ingredient in the production of aflatoxins, a group of common and extremely hazardous secondary metabolites, the most potent naturally occurring toxic and hepatocarcinogenic compounds [4]. About 4.5 billion people are affected by uncontrolled amounts of aflatoxin in developing countries; aflatoxicosis is ranked sixth among the 10 most important health risks identified by Williams et al. [5].

Despite the recent introduction of new antifungal drugs and synthetic preservatives, the application of synthetic antifungal agents has led to a notable increase in drug resistance [6]. In this context, investigators are looking for effective antimicrobial agents to control disease and food contamination. The antimicrobial properties of plant products have been recognized and used for antifungal agents since ancient times in China. Among the different groups of plant products, essential oils are especially recommended as one of the most promising groups of natural products for the formulation of safer antifungal agents [7]. Majority of the essential oils are classified as Generally Recognized as Safe (GRAS) and have low risk for resistance development in pathogenic microorganisms [8].

*Anethum graveolens* L. (dill), an important member of the Umbelliferae family native to southwest Asia or southeast Europe, is widely used for flavoring foods and beverages, and for the treatment of many pathological conditions such as disease of the uterus, cervical ectropio, flatulence, indigestion, stomachache, colic, and gas in the intestinal tract [9]. Dill has been reported to possess antibacterial [10], antihyperlipidemic, and antihypercholesterolemic [11] properties. As a traditional medicine, dill increases milk production and promotes menstruation [12]. Based on our previous work, dill oil can be a potential source of eco-friendly antifungal drugs and food preservatives [13], [14]. However, to our knowledge and according to a literature survey, there are no available reports on the underlying mechanism of antifungal action of dill oil against *A. flavus*. Thus, a further exploration of the subject is necessary.

In the present study, the plasma membrane and mitochondria in *A. flavus* were taken as potential targets for antifungal activity. To determine the exact target of dill oil in the plasma membrane, the effect of the oil on a lesion in the plasma membrane and the content of ergosterol were determined. The effects of dill oil on various markers of mitochondrial activity, such as mitochondrial membrane potential (MMP), acidification of external medium, mitochondrial ATPase, mitochondrial dehydrogenases, and reactive oxygen species (ROS) production, were investigated in *A. flavus*.

## Materials and Methods

### Chemicals

Propidium iodide (PI), menadione, rhodamine 123 (Rh123), (2,3)-bis-(2-methoxy-4-nitro-5-sulphenyl)-(2H)-tetrazolium-5-carboxanilide (XTT), L-cysteine (Cys), and 2′, 7′-dichloro fluorescin–diacetate (DCFH-DA) were purchased from Sigma Chemical Co. (St. Louis, MO, USA).

### Plant material

The seed parts of *A. graveolens* plants were harvested from the Xinjiang Uyghur Autonomous Region of China in May 2008 (No specific permits were required for the described field studies or for the collection of plant material). The plant material was initially identified by its morphological features and was finally confirmed by the corresponding author. Voucher specimen no. 581 was deposited at the herbarium of the Institute of Traditional Chinese Medicine & Natural Products, Wuhan University School of Pharmaceutical Sciences.

### Isolation of the essential oil

A total of 200 g air-dried seeds were grounded using a mill (FW-100, Taisite Instrument Co., Ltd, Tianjin, China). The grounded seeds were passed through a mesh screen to obtain a uniform powder (less than 0.25 mm), which was then subjected to hydrodistillation for approximately 5 h using a Clevenger-type apparatus (SS85-1000, Shenshi Chemical Engineering Co., Ltd., Wuhan, China). The essential oil yield was 3.5% (v/w). It was dried over anhydrous sodium sulfate. After filtration, it was stored in airtight sealed glass vials covered with aluminum foil at approximately 4°C for further testing.

### Fungal strains used

*A. flavus* CCAM 080001, with an MIC (minimum inhibitory concentration) of 2 µl/ml reported from our previous work [13], was obtained from the Culture Collection of State Key Laboratory of Agricultural Microbiology (CCAM) in China. The fungal strain cultures were maintained on a potato dextrose agar (PDA) slant at 4°C. The old cultures were transferred to a fresh slant every two months to avoid a decline in strain viability.

### Determination of lesion of plasma membrane

The studies on membrane damage were tested following the procedure described previously, but with slight modifications [15]. A spore suspension of *A. flavus* was obtained from its 3-day-old cultures, which were harvested by adding 5 ml PBS with 2% (w/v) D-glucose (PBS-2%G) to each petri dish and gently scraping the mycelial surface three times with a sterile L-shaped spreader to free the spores. The spore suspension of *A. flavus* containing 4×10^6^ spores/ml adjusted by a hemocytometer was then added into each glass tube. A requisite amount of the dill oil was added in the tubes to obtain 0.25, 0.5, 1.0, 1.5, and 2.0 ìl/ml concentrations. Samples without any oil treatment were considered as controls. The mixtures were then incubated for 12 h at 28±2°C in an incubator shaker. The cells were washed and resuspended in 0.5 ml PBS, and stained with a final concentration of 1 µg/ml PI solution in PBS for 30 min at room temperature. All the incubations were carried out in the dark. Unstained cell suspensions were always included as autofluorescence controls. For each sample, a Scattergram analysis was performed to evaluate morphological changes, and the percentage of PI-positive cells was determined using flow cytometer (Epics Altra, Beckman Coulter, Miami, USA) at PMT4 channel (620 nm). The results were analyzed using Expo32 v1.2 software. All tests were performed in triplicate.

### Determination of ergosterol content in the plasma membrane

Ergosterol content in the plasma membrane of *A. flavus* was measured by our previously published method [16]. An amount of 100 µl containing 10^7^ spores/ml (the spore population was counted using a hemocytometer) of *A. flavus* spore suspension was inoculated in a Potato Dextrose Broth (PDB) medium containing 0.25, 0.5, 0.75, and 1.0 µl/ml of dill oil (the failure of mycelia generation at 2.0 µl/ml) for four days at 28±2°C. Samples without any oil treatment were considered as controls. After incubation, mycelia was harvested and washed twice with distilled water. The net wet weight of the cell pellet was determined. Five milliliters of 25% alcoholic potassium hydroxide solution was added to each sample and vortex mixed for 2 min (TS-1, Kylin-Bell Lab Instruments Co., Ltd., Shanghai, China), followed by incubation at 85°C for 4 h. Sterols were extracted from each sample by adding a mixture of 2 ml sterile distilled water and 5 ml *n*-heptane. The mixture was then sufficiently mixed by vortex (TS-1, Kylin-Bell Lab Instruments Co., Ltd., Shanghai, China) for 2 min allowing the layers to separate for 1 h at room temperature. The *n*-heptane layer was analyzed using scanned spectrophotometry (UV-1700, Shimadzu, Tokyo, Japan) between 230 and 300 nm. The presence of ergosterol (at 282 nm) and the late sterol intermediate 24(28) dehydroergosterol (at 230 and 282 nm) in the *n*-heptane layer led to a characteristic curve. Ergosterol amount was calculated as a percentage of the wet weight of the cells, and was based on the absorbance and wet weight of the initial pellet. The calculated formula of the ergosterol amount is as follows:
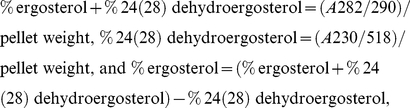
where 290 and 518 are the E values (in percentages per cm) determined for crystalline ergosterol and 24(28) dehydroergosterol, respectively, and pellet weight is the net wet weight (g).

### Determination of the MMP

The change of MMP in *A. flavus* after treatment with dill oil was analyzed using fluorescent dye Rh123 through a confocal laser scanning microscope (Leica TCS-SP, Leica, Heidelberg, Germany) and FACScan flow cytometer (Epics Altra, Beckman Coulter, Miami, USA) [17]. The fungal cell suspension obtained in PBS-2%G was adjusted to 4×10^6^ spore/ml and treated with dill oil in the following final concentrations: 0.25, 0.5, 1.0, and 2.0 µl/ml. Samples without any oil treatment were considered as controls. Rh123 was added into the mixture with a final concentration of 100 ng/ml for 12 h in the dark at 28°C. After incubation, the fungal cells were centrifuged at 5000×*g* for 5 min and washed twice with PBS. The pellet was resuspended in 0.5 ml PBS. Each sample was then observed with a confocal laser scanning microscope (Leica TCS-SP, Leica, Heidelberg, Germany). The fluorescence intensities were measured using a flow cytometer (Epics Altra, Beckman Coulter, Miami, USA). Results were expressed as having a fluorescence intensity of Rh123 with excitation at 488 nm and emission at 525 nm. All tests were performed in triplicate.

### Determination of acidification of external medium

The proton pumping activity of *A. flavus* was detected by monitoring the glucose-induced acidification of the external medium by detecting the pH as previously reported method with slight modifications [18]. The spore suspension containing 10^7^ spore/ml concentrations in PBS-2%G was inoculated in each Erlenmeyer flask. The flasks were then incubated for 48 h at 28±2°C. Cultures containing mycelia after 48 h were filtered through filter paper (DX102, Xinhua Paper Co., Ltd., Hangzhou, China) and then washed twice with distilled water. Approximately 1.0 g wet weight of the washed mycelia was suspended in 40 ml solution containing 50 mM KCl. The suspensions were then incubated at 4°C for 18 h for glucose starvation. The oil was added to the suspension to achieve final concentrations of 0.25, 0.5, 1.0, 2.0, and 4.0 µl/ml. The volume was adjusted to 45 ml with the addition of 50 mM KCl. Samples without any oil treatment were considered as controls. The mixtures were incubated for 10 min at room temperature, and the mycelia were filtered using filter paper (DX102, Xinhua Paper Co., Ltd., Hangzhou, China). A 10% glucose solution of 20 ml was added to the mycelia to induce medium acidification. The value of the external pH was checked using a digital pH meter (PHS-3TC, Shanghai Tianda Instrument Co., Ltd, Shanghai, China) at 0, 10, 20, 30, 40, 50, and 60 min.

### Preparations of mitochondria

The mitochondria of *A. flavus* were isolated using the previously described method with some modifications [19]. An amount of 100 µl containing 10^7^ spores/ml of *A. flavus* spore suspension was inoculated in PDB medium containing 0, 0.25, 0.5, 0.75, and 1.0 µl/ml of dill oil (the failure of mycelia generation at 2.0 µl/ml) for four days at 28±2°C. After incubation, mycelia was harvested and washed twice with distilled water. The net wet weight of the cell pellet was determined. Each approximately 2 g wet weight of mycelium was resuspended in 10 volumes of isolation buffer (10 mM Tris-HC1, 1 mM EDTA, 0.5 M mannitol, pH 7.0), and then disrupted in a high-speed homogenizer (XHF-D, Xinzhi Scientific Biotechnology Co., Ltd., Ningbo, China) for 45 s twice. The homogenate was centrifuged at 2000×*g* for 10 min at 4°C to remove mycelial fragments and conidia. The supernatant was carefully removed from the loose pellet and centrifuged at 12000×*g* for 40 min at 4°C. The pellet containing the mitochondria was washed in an isolation buffer and re-centrifuged using the same rotor for 20 min. The final mitochondrial pellet was resuspended in 1 ml isolation buffer and maintained at 4°C until use.

### Determination of mitochondrial ATPase activity

The mitochondrial ATPase activity in dill oil-treated fungal cells was detected using a Micro-ATPase Assay Kit obtained from the Institute of Biological Engineering of Nanjing Jianchen (Nanjing, China) following the manufacturer's protocol. Protein content was determined as described by Bradford [20], and bovine serum albumin was used as a standard. The optical density (OD) value was determined at 636 nm after 5 min. Next, 20 nM inorganic phosphate was used as a control. One unit of ATPase activity was defined as 1 µmol inorganic phosphorus catalyzed by this enzyme in 1 mg protein for 1 h (µmolPi/mgpro/h).

### Determination of mitochondrial dehydrogenases activity

The change of mitochondrial dehydrogenases in *A. flavus* after treatment with dill oil was measured using the XTT method [21], [22]. Briefly, 200 µl of 2×10^6^ spore/ml was added to 96-well flat-bottom microplates (Corning, Corning Incorporated, New York, USA) and incubated with different concentrations of dill oil (0.0313, 0.0625, 0.125, 0.25, 0.5, 1.0, 2.0, and 4.0 µl/ml). Samples without any oil treatment were considered as controls. After 24 h of incubation at 28°C, 50 µl aliquots stock XTT with menadione was added to the wells in order to obtain a final concentration of 50 µg/ml XTT and 25 µM menadione. The optical densities at 450 nm (OD_450_) were determined directly using a 96-well scanner (KHB ST-360, Experimental System Co., Ltd. Shanghai, China) after 2 h of exposure to XTT. The XTT assay was performed in triplicate.

### Determination of endogenous ROS production

Endogenous ROS levels of *A. flavus* were detected by a fluorometric assay using the fluorescent dye DCFH-DA as a ROS indicator as previously reported with some modifications [23]. Fungal cell suspension obtained was adjusted to 4×10^6^ spore/ml and exposed to dill oil with final concentrations of 0.25, 0.5, 1.0, and 2.0 µl/ml for 12 h. Samples without any oil treatment were considered as controls. At the end of the treatments, DCFH-DA was added into the mixture with a final concentration of 10 µM for 4 h at 28°C. After incubation, the fungal cells were centrifuged at 5000×*g* for 5 min and washed twice with PBS; the pellet was resuspended in 0.5 ml PBS. Fluorescence intensities of the resuspended cells were quantified via FACScan flow cytometer (Epics Altra, Beckman Coulter, Miami, USA) using excitation and emission wavelengths of 485 and 535 nm, respectively. Additionally, these experiments were performed with the presence of the antioxidant Cys at concentrations of 80 mM. Results were corrected by subtracting the fluorescence value of dill oil ± antioxidant in the corresponding concentration without cells but with DCFH-DA. All tests were performed in triplicate.

### Statistical analysis

All data are reported as means ± standard deviations. The significant differences between mean values were determined using Duncan's Multiple Range test (*p*<0.05) following one-way ANOVA. The statistical analysis was performed using a statistical software (SPSS, 13.0; Chicago, USA).

## Results

### Effect of dill oil on lesion of plasma membrane

**Figure 1:**
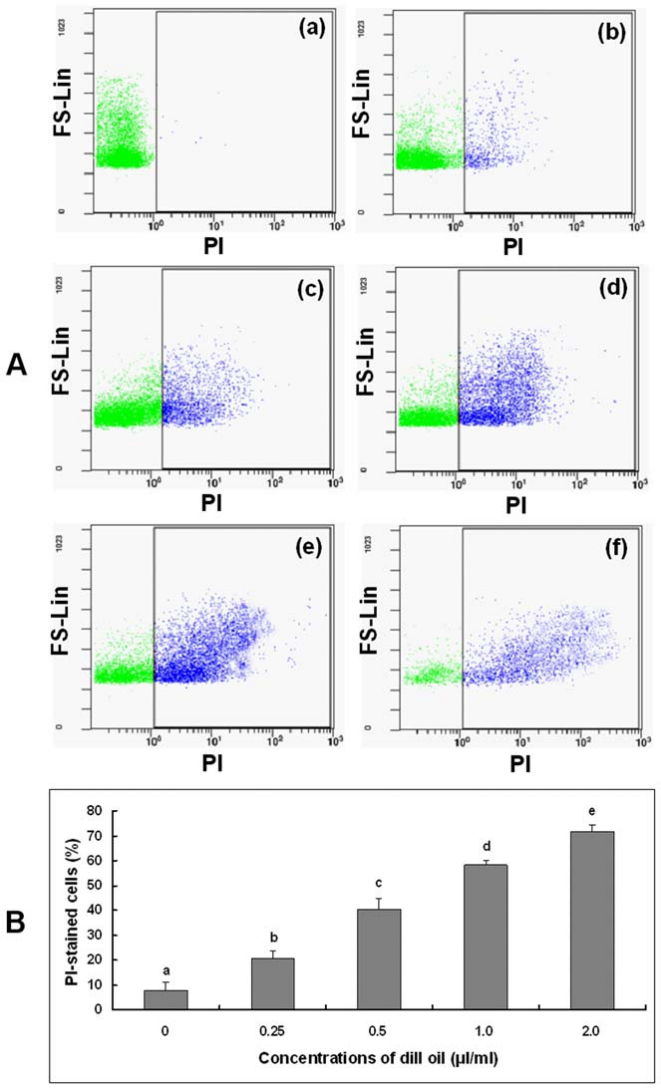
Efficacy of dill oil on the lesion of plasma membrane of *A. flavus* cells. (A) Sequence of density plots exhibiting *A. flavus* cell size (forward scatter, FS-Lin) analyzed by flow cytometry at PMT4 channel (620 nm), and the respective percentages of PI-stained cells (right quadrant) for a series of samples treated with increasing concentrations of dill oil. (a) autofluorescence of non-treated cells; (b) fluorescence of non-treated cells stained with 1 µg/ml PI for 30 min; (c–f) cells treated with dill oil at 0.25 µl/ml (c), 0.5 µl/ml (d), 1.0 µl/ml (e), 2.0 µl/ml (f). (B) Effect of dill oil on percentage of PI-stained *A. flavus* cells analyzed by flow cytometry for 12 h and compared with an untreated control. Significant differences (*p*<0.05) between means are indicated by letters above the histogram bars. Values are means (n = 3) ± standard deviations.

The investigation relied on the flow cytometric analyses of PI-stained cells to analyze the antifungal activity of dill oil on *A. flavus* cells. The results of staining *A. flavus* cells with PI are presented in Figure 1. Substantial morphological changes were observed on a scattergram of *A. flavus* cells after 12 h incubation at 0.25, 0.5, 1.0, and 2.0 ìl/ml concentrations (Figure 1A). The sequence of density plots showed different cell size (forward scatter, FS Lin) after treatment of serial concentrations of dill oil. Some marked morphological alterations were observed in Figure 1A(c–f) when compared with untreated cells. Figure 1B shows that the percentage of PI-stained *A. flavus* cells detected by flow cytometry after exposure to serial concentrations of dill oil for 12 h, relative to control without dill oil. As shown in Figure 1B, PI was unable to penetrate the cells with intact plasma membranes, while cells exposed to different concentrations of dill oil with injured membranes were stained by PI with percentages ranging from 18.7% to 70.3%.

### Effect of dill oil on plasma membrane ergosterol content

**Figure 2:**
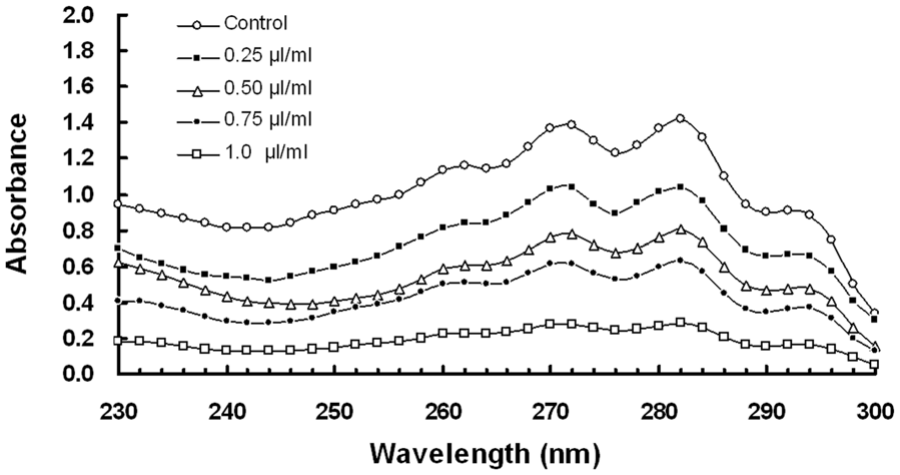
Inhibition of ergosterol biosynthesis in *A. flavus* by dill oil. UV spectrophotometric sterol profiles of *A. flavus* cells treated with dill oil and in comparison with those of an untreated control.

The efficacies of dill oil on the ergosterol content in the plasma membrane of *A. flavus* are shown in Figure 2. The total ergosterol content was determined at 0, 0.25, 0.5, 0.75, and 1.0 µl/ml concentrations of dill oil with values of 0.609±0.006%, 0.443±0.005%, 0.314±0.005%, 0.275±0.003%, and 0.125±0.003%, respectively (data not presented). Ergosterol content (at 282 nm) in the plasma membrane of *A. flavus* was significantly inhibited by the different concentrations of essential oil. A dose-dependent decrease in ergosterol production was observed when isolates were grown in the presence of dill oil. After the incubation of *A. flavus* at 0.25, 0.5, and 0.75 µl/ml concentrations of dill oil, a reduction percentage of the ergosterol content in the plasma membrane compared with the control was observed at 27.3% for 0.25 µl/ml, 48.4% for 0.5 µl/ml, and 54.8% for 0.75 µl/ml. *A. flavus* cells growing in the presence of 1.0 µl/ml concentrations of the dill oil showed the highest inhibition to ergosterol with a value of 79.4% compared with the control.

### Effect of dill oil on the MMP

**Figure 3:**
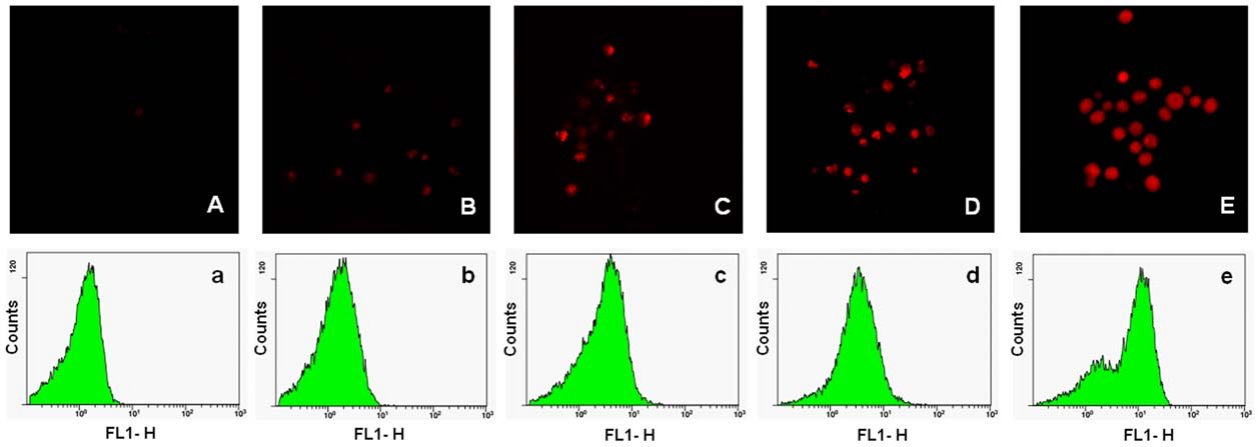
Efficacy of dill oil on MMP of *A. flavus* cells. The measurements were performed by confocal laser scanning microscope (top panels) and flow cytometry (bottom panels) stained with Rh123. (A, a) Control, (B, b) Dill oil at 0.25 µl/ml, (C, c) Dill oil at 0.5 µl/ml, (D, d) Dill oil at 1.0 µl/ml, (E, e) Dill oil at 2.0 µl/ml.

MMP is a very sensitive indicator of the energy-coupling condition of mitochondria [24]. Thus, MMP can be determined using a confocal laser scanning microscope and flow cytometry using the fluorescent dye Rh123, a cell permeable cationic dye, which preferentially enters the mitochondria based on the highly negative MMP. As shown in Figure 3, treatment with dill oil caused MMP degradation in a dose-dependent manner. The different concentrations of dill oil visibly decreased the fraction of cells with low fluorescence and increased the fraction of cells with high fluorescence (the top panels of Figure 3). When *A. flavus* cells were exposed to 0.25, 0.5, 1.0, and 2.0 µl/ml concentrations of oil, the MMPs of cells, calculated from the mean fluorescence of all tested cells, were 2.03±0.25, 4.37±0.15, 9.27±0.21, 14.27±0.25, and 20.23±0.55 mV, respectively (data not shown). Based on the abovementioned results, dill oil can reduce the MMP in *A. flavus* cells on concentration-dependent.

### Effect of dill oil on acidification of external medium

**Figure 4:**
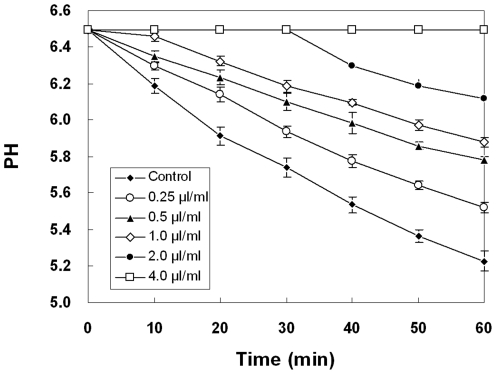
Efficacy of different concentrations of dill oil on the glucose-dependent acidification of medium in *A. flavus*. Values are means (n = 3) ± standard deviations.

*A. flavus* highly susceptible to dill oil was detected for its ability to pump intracellular protons to the external medium in the presence of various concentrations of dill oil. As shown in Figure 4, dill oil inhibits the glucose-induced reduction in external pH of *A. flavus* in a time- and concentration-dependent fashion. Medium acidification by *A. flavus* was significantly inhibited by dill oil at 0.25, 0.5, and 1.0 µl/ml concentrations after incubation for 10 min. However, at 2.0 and 4.0 µl/ml, the medium acidification was completely inhibited for 30 min, and at 4.0 µl/ml, the medium acidification was completely inhibited at 60 min.

### Effect of dill oil on mitochondrial ATPase activity

**Figure 5:**
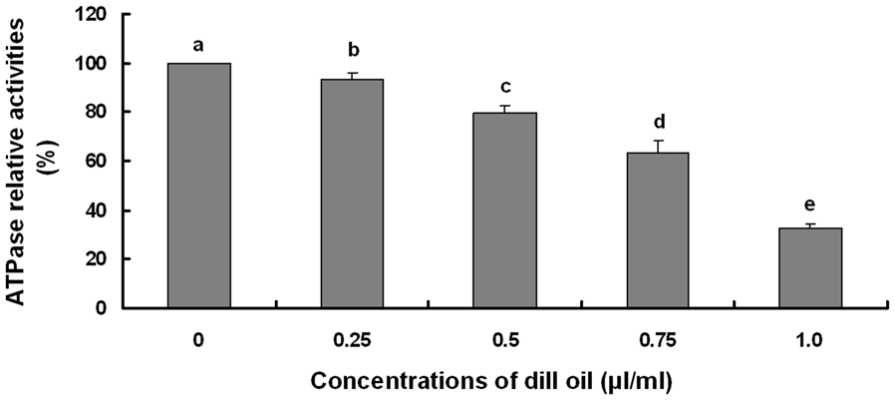
Efficacy of dill oil on the activities of the mitochondrial ATPase of *A. flavus* cells. Significant differences (*p*<0.05) between means are indicated by letters above the histogram bars. Values are means (n = 3) ± standard deviations.

Dill oil exhibited a concentration-dependent inhibitory effect on the activity of the mitochondrial ATPase of *A. flavus* as shown in Figure 5. Four different concentrations of the essential oil were found to be effective in significantly inhibiting the activities of mitochondrial ATPase (*p*<0.05). At concentrations of 0.25, 0.5, 0.75, and 1.0 µl/ml, the ATPase activity was reduced to values around 7%–70%. These results demonstrate that dill oil inhibits the mitochondrial ATPase activity.

### Effect of dill oil on mitochondrial dehydrogenases

**Figure 6:**
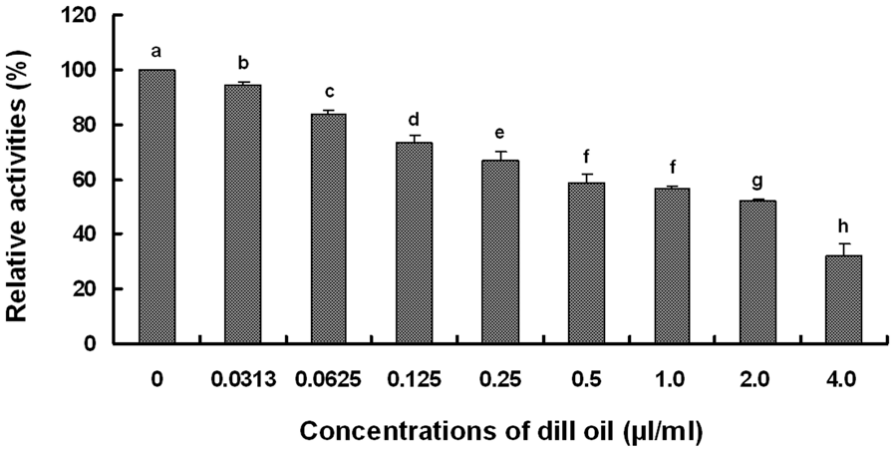
Efficacy of dill oil on the activities of the mitochondrial dehydrogenases of *A. flavus* cells. Significant differences (*p*<0.05) between means are indicated by letters above the histogram bars. Values are means (n = 3) ± standard deviations.

The activities of mitochondrial dehydrogenases exposed to dill oil were determined through colorimetric assay using XTT as substrate, which can be converted into a colorful water-soluble formazan product by mitochondrial dehydrogenases in healthy cells [25]. The efficacy of the dill oil on activities of mitochondrial dehydrogenases are shown in Figure 6. The eight different concentrations of essential oil caused different degrees of inhibition on activities of mitochondrial dehydrogenases (*p*<0.05). The data indicated the dill oil effect on the activity of the mitochondrial dehydrogenases of *A. flavus* in a concentration-dependent manner. Relative activities of mitochondrial dehydrogenases exposed to dill oil at concentrations of 0.0313, 0.0625, 0.125, 0.25, 0.5, 1.0, 2.0, and 4.0 µl/ml compared with control subjects were reduced by 5.59%±1.00%, 16.29%±1.30%, 26.53%±2.49%, 33.25%±3.49%, 41.30%±3.09%, 43.68%±1.43%, 48.01%±0.68%, and 68.19%±4.83%, respectively.

### Effect of dill oil on endogenous ROS production

**Figure 7:**
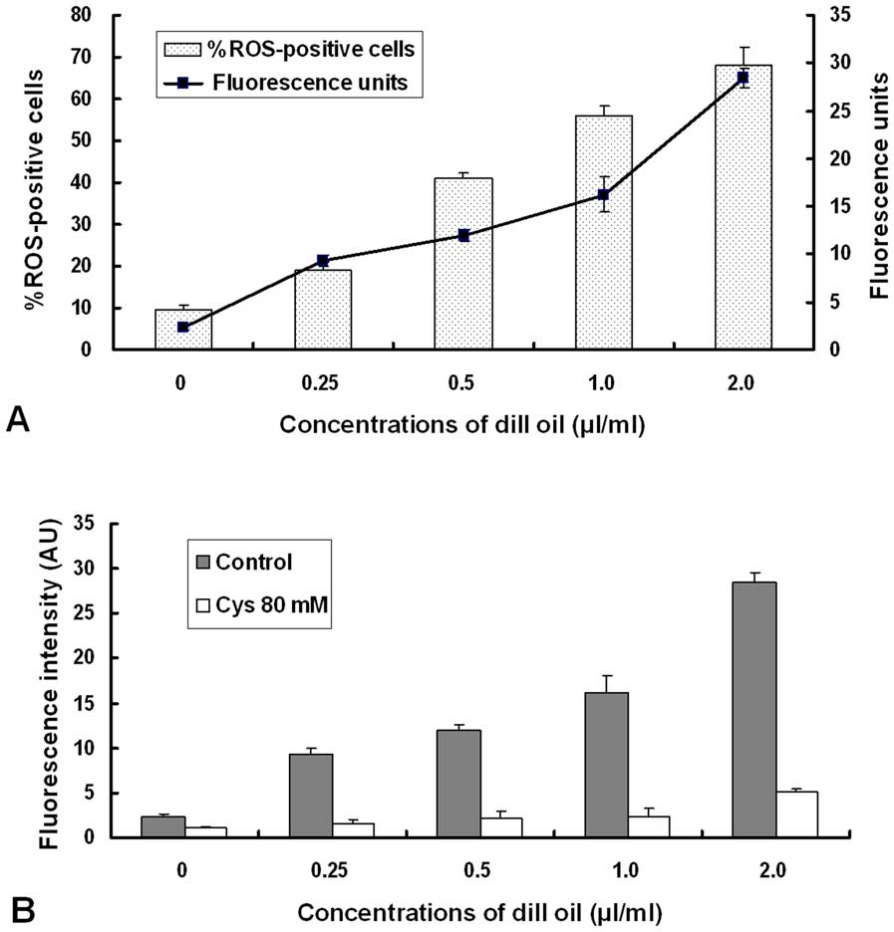
Efficacy of dill oil on the endogenous ROS in *A. flavus* cells detected by flow cytometry. (A) Dill oil-induced increase of endogenous ROS in *A. flavus* cells. (B) Effect of antioxidant Cys on dill oil-induced ROS production in *A. flavus* cells.

The fluorescent dye DCFH-DA, a well-established compound to assay and quantify ROS production, was used to investigate the changes in intracellular ROS level of *A. flavus* cells. As shown in Figure 7A, an increase in the fluorescence intensity was detected in *A. flavus* cells incubated with different concentrations of dill oil for 12 h in a dose-dependent manner. Compared with the control, at a concentration of 0.25, 0.5, 1.0, and 2.0 µl/ml of dill oil, the promotion of the fluorescence intensity corresponded to 4.06, 5.19, 7.06, and 12.36 times, respectively. In addition, the percentage of ROS-positive cells clearly increased with the concentration of dill oil (Figure 7A). Dill oil was observed to induce ROS accumulation in *A. flavus* cells.

To investigate whether ROS production is directly involved in the antifungal effect of dill oil and is not merely a secondary effect of dill oil action, the effect of an antioxidant Cys on the net level of ROS production and antifungal activity in dill oil-treated *A. flavus* cells was measured. As shown in Figure 7B, the net ROS production in cells induced by dill oil treatment was evidently depressed by the addition of the antioxidant Cys. With the presence of Cys, the quantity of ROS in cells only increased slightly with the MIC concentration (2.0 µl/ml), which suggests that Cys can arrest the induced ROS production by dill oil. Thus, ROS formation plays a critical role in dill oil-induced killing of *A. flavus* cells.

## Discussion

Essential oils, which are aromatic volatile products of the secondary metabolism of plants, have been applied in natural remedies, perfumes and make-up products, sanitary products, in dentistry, in agriculture, as food preservatives, and as additives [26]. Generally, whole essential oils have greater antifungal activity due to a synergistic effect with some active components; thus, they are more promising in commercial application than single compounds. The chemical compositions of dill oil used in the present study were previously determined by our laboratory. Using gas chromatography coupled with mass spectrometry, the major components detected in dill oil are carvone (41.5%), limonene (32.6%), and apiol (16.8%). The antifungal activities exhibited by dill oil might be attributed to these major components.

The plasma membrane plays a vital role in maintaining a homeostatic environment, exchanging materials, and transferring energy and information in the cell to keep cells healthy and alive. Furthermore, the key role of mitochondria is to provide a myriad of services to the cell, including energy production, pH homeostasis, redox, calcium buffering, regulation of level of second messengers, production and transmission of a transmembrane potential, and regulation of apoptosis [27], [28]. Based on the present study, the plasma membrane and the mitochondria are the antifungal targets of dill oil.

Dead or dying cells with injured membranes can incorporate PI, which is a nucleic acid-binding fluorescent probe [29]. PI is one of the most popular fluorescent probes used in assessing the effect of drugs on plasma membranes [15]. The observed dose-dependent antifungal activity of dill oil on *A. flavus* cells with a severe lesion of the plasma membrane results from the direct damage to the membrane, rather than from metabolic impairment leading to secondary membrane damage. Dill oil may change the plasma membrane structure, indicating the absence of the integrity of fungal cells. Thus, the dill oil acts by primary lesion of the membrane. Similar types of results were also reported by some investigators [15], [30], [31].

To ensure the dill oil target in the plasma membrane, the effect of dill oil on the amount of ergosterol was assessed. Ergosterol is specific to fungi and is the major sterol component of the fungal cell membrane. It is also responsible for maintaining cell function and integrity [32]. As mentioned earlier, Kelly et al. [33] states that the primary action mechanism by which azole antifungal drugs inhibit fungal cell growth is the disruption of normal sterol biosynthetic pathways resulting in a decrease of ergosterol biosynthesis. Some previous studies have exhibited that natural and synthetic drugs can cause a considerable reduction in the quantity of ergosterol [15], [31], [34]–[36]. In our experiment, ergosterol content, which is an absolute measurement, was determined using previously described methods [16]. This sterol quantitation method is indicative of the ergosterol and 24(28)dehydroergosterol contents based on the exclusive spectral absorption pattern produced between 230 and 300 nm by extracted sterols. Thus, the important target in the plasma membrane was determined by quantitating the total intracellular ergosterol production in cells grown in increasing concentrations of dill oil. Our observations revealed that dill oil can induce a considerable impairment of the ergosterol biosynthesis by *A. flavus*. Hence, the plasma membrane is an important antifungal target of dill oil.

The mitochondria maintain a MMP across their inner membrane in healthy cells due to an electrochemical gradient maintained through electron transport chain [37]. Oxidative phosphorylation of mitochondria is required for the production of energy (ATP) in healthy cells, in which MMP plays a very critical role in mitochondria [38]. Inhibitors of mitochondrial electron transport reduce MMP via inhibitors of the proton-pumping function of the respiratory chain, leading to the reduction of ATP production and cell death [39]. The disturbance in the MMP has also been considered a signal for the onset of apoptosis [40]. In the present study, the Rh123 was used to monitor the changes in MMP by dill oil. The Rh123 is a kind of cationic and lipophilic dye that can permeate into the negatively charged mitochondria and reflect the MMP. After 12 h of treatment, a significant hyperpolarization in the MMP in a concentration-dependent was observed on the mitochondria with different concentrations of dill oil. The present results demonstrate that dill oil affects the functional integrity of mitochondria. Although several studies have focused on the effects of antifungal agents on the MMP of yeast [39], [41], the ability of antifungal agents to affect the MMP in *A. flavus* has not been demonstrated. It should be noted according to our experiment that a rare Rh123 could permeate into the cells exposed to the oil for a short amount of time (not longer than 1 h). However, a massive Rh123 can permeate into the cells when exposed for a longer period of time (no less than 4 h), which may be due to the few lipophilic components of the filamentous fungus in the cell membrane or cell wall. Hence, it is more difficult for the lipophilic dye to permeate into *A. flavus* cells than into yeast.

Dill oil has been shown to affect the functioning of the plasma membrane ATPase, as suggested by the inhibition of medium acidification. In addition, dill oil appears to inhibit the mitochondrial ATPase, affecting medium acidification indirectly by reducing or depleting the large amounts of cellular ATP required to fuel the plasma membrane ATPase. Acidification of the medium may occur initially through the proton-pumping action of the plasma membrane ATPase. Cells can recover by pumping intracellular protons out of the cell via the plasma membrane ATPase, consuming large amounts of ATP after glucose is added to non-growing cells [42]. Nevertheless, a decrease in ATP to levels below those necessary for normal metabolic functions is a key pathway leading to cell death. Thus, dill oil essentially inhibits mitochondrial ATPase, decreasing a major source of ATP, which results in a suppression of plasma membrane ATPase and a decrease in the pH of the external medium.

The energy needed to drive plasma membrane ATPase was being affected; therefore, mitochondrial ATPase activity was further detected as a likely essential target for dill oil, which could significantly inhibit mitochondrial ATPase activity in fungal cells. Mitochondrial ATPase normally functions to produce ATP, but when free of the driving force of the electron transport chain, it can function in reverse and hydrolyze ATP [43]. Thus, the activity of mitochondrial ATPase can be measured for their ability to hydrolyze ATP. The mitochondrial ATPase plays a critical role in energy generation, and inhibition would contribute to an evident degradation in ATP content, which would indirectly inhibit the plasma membrane proton-pumping ATPase and account for the inhibition of acidification [42]. Finally, a reduction in ATP content leads to the dysfunction of normal cells. Dill oil inhibited an isolated mitochondrial ATPase in a dose-dependent manner at different concentrations, which manifests the idea of mitochondrial ATPase being a physiologically relevant target of dill oil in *A. flavus*. Similar types of results were also reported by Lunde et al. [44] and Castelli et al. [42].

The activities of the mitochondrial dehydrogenases exposed to dill oil were detected by XTT, which can be metabolically decreased by mitochondrial dehydrogenases in viable cells [45]. Mitrochondrial dehydrogenases are key catalyzing enzymes in the biosynthesis of ATP, the activity of which was examined in this assay including the activities of various dehydrogenases in the mitochondrion, such as lactate dehydrogenase (LDH), malate dehydrogenase (MDH), and succinate dehydrogenase (SDH) [39]. LDH is a kind of enzyme that catalyzes the conversion of lactate into pyruvate, the last step in anaerobic glycolysis, which is a vital step in energy production in cells. MDH catalyzes the interconversion of malate to oxaloacetate in the tricarboxylic acid (TCA) cycle. SDH catalyzes the oxidation of succinate to fumarate in the TCA cycle and transfer the electrons from succinate to ubiquinol [46]. Majority of earlier reports on the activities of mitochondrial dehydrogenases focused on yeast cells, whereas Meletiadis et al. developed a colorimetric assay for antifungal susceptibility testing of filamentous fungi based on XTT as substrate [21]. Our observations revealed that activities of the mitochondrial dehydrogenases are evidently inhibited by dill oil, which may interfere with the citric acid cycle and inhibit the synthesis of ATP in the mitochondria of *A. flavus*.

Mitochondria are widely known as organelles inside the cells of higher organisms that represent a major source of ROS. ROS production is commonly caused by some cellular stresses such as irradiation and cytotoxic drugs, leading to growth inhibition and the death of mammalian cells [47]. ROS appears to be important in the assay for cell viability resulting in enzyme inactivation, membrane disruption, and cell death [48]. Moreover, ROS accumulation is considered one of the primary biochemical hallmarks for apoptosis, which promote morphological changes, nuclear fragmentation, chromatin condensation, cellular swelling, and phosphatidylserine externalization [49]. DCFH-DA is commonly used to detect oxidative stress in cells due to the high sensitivity of fluorescence-based assays [50]. In the assay for the detection of ROS, an increase in the amount of ROS was apparent after different doses of dill oil treatment. The addition of antioxidant Cys readily prevented ROS production, indicating that ROS may be an important mediator for the exhibition of the antifungal effects of dill oil. In recent research, similar observations demonstrated that the antifungal action of many antifungal agents is related with the induction of ROS formation in fungi, especially in *Candida* species [39], [48], [51], [52]. However, to date, little has been documented about antifungal agents involved in the induction of ROS formation in filamentous fungi, which was reported in *Rhizoctonia*, *Fusarium*, and *Aspergillus* species [51], [53]–[55]. Hence, ROS accumulation is a common pathway underlying cellular damage induced by different types of stresses including exposure to dill oil.

Evidently, there is a causal link between the antifungal action of dill oil and the abnormal cells, including plasma membrane and mitochondrial dysfunction. The flow cytometry data and the impairment in the biosynthesis of ergosterol in this study confirm the dill oil-induced lesion of the plasma membrane structure as the antifungal target of oil. Afterwards, the lesion of the plasma membrane may be associated with ion loss, reduction of membrane potential, collapse of the proton pump, and consumption of the ATP pool [39]. Normally, the higher MMP is accompanied by more efficient electron transportation, which would lead to leakage of more electrons to generate ROS. Previous work revealed that the generation of ROS is exponentially dependent on MMP [56], which is in agreement with our result that both MMP and endogenous ROS were conspicuously augmented in *A. flavus* cells after dill oil treatment. In addition, the inhibition of dill oil on mitochondrial ATPase resulted in an evident degradation in ATP contents, which would indirectly inhibit the plasma membrane proton-pumping ATPase and account for the inhibition of acidification. The inhibitory effect of oil on mitochondrial dehydrogenases also reduced the ATP synthesis, both of which caused ATP depletion in mitochondria to levels below those necessary for normal metabolic functions. These dysfunctions of mitochondria enhanced the accumulation of ROS in *A. flavus* cells, thereby leading to cell death by oxidative damage to a biomacromolecule or mediation of apoptosis.

In conclusion, the antifungal activity of dill oil results from its ability to disrupt the permeability barrier of the plasma membrane and from the mitochondrial dysfunction-induced ROS accumulation in *A. flavus*. The current study and earlier research may provide a detailed understanding of dill oil as an antifungal agent.

## References

[1] Cleveland TE, Yu J, Fedorova N, Bhatnagar D, Payne GA (2009). Potential of *Aspergillus flavus* genomics for applications in biotechnology.. Trends Biotechnol.

[2] Denning DW, Riniotis K, Dobrashian R, Sambatakou H (2003). Chronic cavitary and Fibrosing pulmonary and pleural aspergillosis: Case series, proposed nomenclature change, and review.. Clin Infect Dis.

[3] Maertens J, Raad I, Petrikkos G, Boogaerts M, Selleslag D (2004). Efficacy and safety of caspofungin for treatment of invasive aspergillosis in patients refractory to or intolerant of conventional antifungal therapy.. Clin Infect Dis.

[4] Squire RA (1981). Ranking animal carcinogens: a proposed regulatory approach.. Science.

[5] Williams JH, Phillips TD, Jolly PE, Stiles JK, Jolly CM (2004). Human aflatoxicosis in developing countries: a review of toxicology, exposure, potential health consequences, and interventions.. Am J Clin Nutr.

[6] White TC, Marr KA, Bowden RA (1998). Clinical, cellular and molecular factors that contribute to antifungal drug resistance.. Clin Microbiol Rev.

[7] Varma J, Dubey NK (2001). Efficacy of essential oils of *Caesulia axillaris* and *Mentha arvensis* against some storage pests causing biodeterioration of food commodities.. Int J Food Microbiol.

[8] Cardile V, Russo A, Formisano C, Rigano D, Senatore F (2009). Essential oils of *Salvia bracteata* and *Salvia rubifolia* from Lebanon: Chemical composition, antimicrobial activity and inhibitory effect on human melanoma cells.. Journal of Ethnopharmacology.

[9] Editorial Committee of National Chinese Medical Manage Bureau (1999). Chinese Herb, Vol. 15.

[10] Rafii F, Shahverdi AR (2007). Comparison of essential oils from three plants for enhancement of antimicrobial activity of nitrofurantoin against enterobacteria.. Chemotherapy.

[11] Yazdanparast R, Alavi M (2001). Antihyperlipidaemic and antihypercholesterolaemic effects of *Anethum graveolens* leaves after the removal of furocoumarins.. Cytobios.

[12] Monsefi M, Ghasemi M, Bahaoddini A (2006). The effects of *Anethum graveolens* L. on female reproductive system.. Phytother Res.

[13] Tian J, Ban XQ, Zeng H, Huang B, He JS (2011). *In vitro* and *in vivo* activity of essential oil from dill (*Anethum graveolens* L.) against fungal spoilage of cherry tomatoes.. Food Control.

[14] Zeng H, Tian J, Zheng HC, Ban XQ, Zeng JS (2011). *In vitro* and *in vivo* activities of essential oil from the seed of *Anethum graveolens* L. against *candida* spp.. Evid Based Complement Alternat Med.

[15] Pinto E, Vale-Silva L, Cavaleiro C, Salgueiro L (2009). Antifungal activity of the clove essential oil from *Syzygium aromaticum* on *Candida*, *Aspergillus* and *dermatophyte* species.. J Med Microbiol.

[16] Tian J, Huang B, Luo XL, Zeng H, Ban XQ (2012). The control of *Aspergillus flavus* with *Cinnamomum jensenianum* Hand.-Mazz essential oil and its potential use as a food preservative.. Food Chem.

[17] Tang XQ, Feng JQ, Chen J, Chen PX, Zhi JL (2005). Protection of oxidative preconditioning against apoptosis induced by H_2_O_2_ in PC12 cells: mechanisms via MMP, ROS, and Bcl-2.. Brain Res.

[18] Manavathu EK, Dimmock JR, Vashishtha SC, Chandrasekar PH (1999). Proton-Pumping-ATPase-targeted antifungal activity of a novel conjugated styryl ketone.. Antimicrob Agents Chemother.

[19] Rowlands RT, Turner G (1974). Physiological and biochemical studies of nuclear and extranuclear oligomycin-resistant mutants of *Aspergillus nidulans*.. Mol Gen Genet.

[20] Bradford MM (1976). A rapid and sensitive method for the quantitation of microgram quantities of protein utilizing the principle of protein-dye binding.. Anal Biochem.

[21] Meletiadis J, Mouton JW, Meis JFGM, Bouman BA, Donnelly JP (2001). Colorimetric assay for antifungal susceptibility testing of *Aspergillus* species.. J Clin Microbiol.

[22] Deak E, Wilson SD, White E, Carr JH, Balajee SA (2009). *Aspergillus terreus* accessory conidia are unique in surface architecture, Cell Wall Composition and Germination Kinetics.. Plos One.

[23] Helmerhorst EJ, Troxler RF, Oppenheim FG (2001). The human salivary peptide histatin 5 exerts its antifungal activity through the formation of reactive oxygen species.. Proc Natl Acad Sci U S A.

[24] Brand MD, Chien LF, Ainscow EK, Rolfe DF, Porter RK (1994). The causes and functions of mitochondrial proton leak.. Biochim Biophys Acta.

[25] Roehm NW, Rodgers GH, Hatfield SM, Glasebrook AL (1991). An improved colorimetric assay for cell proliferation and viability utilizing the tetrazolium salt XTT.. J Immunol Methods.

[26] Bakkali F, Averbeck S, Averbeck D, Waomar M (2008). Biological effects of essential oils - A review.. Food Chem Toxicol.

[27] McBride HM, Neuspiel M, Wasiak S (2006). Mitochondria: More than just a powerhouse.. Curr Biol.

[28] Zorov DB, Krasnikov BF, Kuzminova AE, Vysokikh M, Zorova LD (1997). Mitochondria revisited. Alternative functions of mitochondria.. Biosci Rep.

[29] Bauer KD (1993). Quality control issues in DNA content flow cytometry.. Ann N Y Acad Sci.

[30] Cox SD, Mann CM, Markham JL, Bell HC, Gustafson JE (2000). The mode of antimicrobial action of the essential oil of *Melaleuca alternifolia* (tea tree oil).. J App Microbiol.

[31] Pinto E, Pina-Vaz C, Salgueiro L, Goncalves MJ, Costa-de-Oliveira S (2006). Antifungal activity of the essential oil of *Thymus pulegioides* on *Candida*, *Aspergillus* and *dermatophyte* species.. J Med Microbiol.

[32] Rodriguez RJ, Low C, Bottema CD, Parks LW (1985). Multiple functions for sterols in *Saccharomyces cerevisiae*.. Biochim Biophys Acta.

[33] Kelly SL, Lamb DC, Corran AJ, Baldwin BC, Kelly DE (1995). Mode of action and resistance to azole antifungals associated with the formation of 14 alpha-methylergosta-8,24(28)-dien-3 beta,6 alpha-diol.. Biochem Biophys Res Commun.

[34] Arthington-Skaggs BA, Warnock DW, Morrison CJ (2000). Quantitation of *Candida albicans* ergosterol content improves the correlation between *in vitro* antifungal susceptibility test results and *in vivo* outcome after fluconazole treatment in a murine model of invasive candidiasis.. Antimicrob Agents Chemother.

[35] Arthington-Skaggs BA, Lee-Yang W, Ciblak MA, Frade JP, Brandt ME (2002). Comparison of visual and spectrophotometric methods of broth microdilution MIC end point determination and evaluation of a sterol quantitation method for *in vitro* susceptibility testing of fluconazole and itraconazole against trailing and nontrailing *Candida* isolates.. Antimicrob Agents Chemother.

[36] Arthington-Skaggs BA, Jradi H, Desai T, Morrison CJ (1999). Quantitation of ergosterol content: Novel method for determination of fluconazole susceptibility of *Candida albicans*.. J Clin Microbiol.

[37] Simbula G, Glascott PA, Akita S, Hoek JB, Farber JL (1997). Two mechanisms by which ATP depletion potentiates induction of the mitochondrial permeability transition.. Am J Physiol.

[38] Kaim G, Dimroth P (1999). ATP synthesis by F-type ATP synthase is obligatorily dependent on the transmembrane voltage.. Embo J.

[39] Wu XZ, Cheng AX, Sun LM, Sun SJ, Lou HX (2009). Plagiochin E, an antifungal bis(bibenzyl), exerts its antifungal activity through mitochondrial dysfunction-induced reactive oxygen species accumulation in *Candida albicans*.. Biochim Biophys Acta.

[40] Wang CX, Youle RJ (2009). The Role of Mitochondria in Apoptosis.. Annu Rev Genet.

[41] Kang K, Fong WP, Tsang PWK (2010). Antifungal activity of baicalein against *Candida krusei* does not involve apoptosis.. Mycopathologia.

[42] Lunde CS, Kubo I (2000). Effect of polygodial on the mitochondrial ATPase of *Saccharomyces cerevisiae*.. Antimicrob Agents Chemother.

[43] Grubmeyer C, Cross RL, Penefsky HS (1982). Mechanism of ATP hydrolysis by beef heart mitochondrial ATPase. Rate constants for elementary steps in catalysis at a single site.. J Biol Chem.

[44] Castelli MV, Lodeyro AF, Malheiros A, Zacchino SA, Roveri OA (2005). Inhibition of the mitochondrial ATP synthesis by polygodial, a naturally occurring dialdehyde unsaturated sesquiterpene.. Biochem Pharmacol.

[45] Buttke TM, McCubrey JA, Owen TC (1993). Use of an aqueous soluble tetrazolium/formazan assay to measure viability and proliferation of lymphokine-dependent cell lines.. J Immunol Methods.

[46] Balietti M, Fattoretti P, Skalicky M, Viidik A, Giorgetti B (2005). The effect of chronic physical exercise on succinic dehydrogenase activity in the heart muscle of old rats.. Biogerontology.

[47] Benhar M, Dalyot I, Engelberg D, Levitzki A (2001). Enhanced ROS production in oncogenically transformed cells potentiates c-Jun N-terminal kinase and p38 mitogen-activated protein kinase activation and sensitization to genotoxic stress.. Mol Cell Biol.

[48] Kobayashi D, Kondo K, Uehara N, Otokozawa S, Tsuji N (2002). Endogenous reactive oxygen species is an important mediator of miconazole antifungal effect.. Antimicrob Agents Chemother.

[49] Pozniakovsky AI, Knorre DA, Markova OV, Hyman AA, Skulachev VP (2005). Role of mitochondria in the pheromone- and amiodarone-induced programmed death of yeast.. J Cell Biol.

[50] Bonini MG, Rota C, Tomasi A, Mason RP (2006). The oxidation of 2′,7′-dichlorofluorescin to reactive oxygen species: A self-fulfilling prophesy?. Free Radic Biol Med.

[51] Mello EO, Ribeiro SFF, Carvalho AO, Santos IS, Da Cunha M (2011). Antifungal Activity of PvD1 Defensin Involves Plasma Membrane Permeabilization, Inhibition of Medium Acidification, and Induction of ROS in Fungi Cells.. Curr Biol.

[52] Yan L, Li MH, Cao YB, Gao PH, Cao YY (2009). The alternative oxidase of *Candida albicans* causes reduced fluconazole susceptibility.. J Antimicrob Chemother.

[53] Cheng JJ, Park TS, Chio LC, Fischl AS, Ye XS (2003). Induction of apoptosis by sphingoid long-chain bases in *Aspergillus nidulans*.. Mol Cell Biol.

[54] Leiter E, Szappanos H, Oberparleiter C, Kaiserer L, Csernoch L (2005). Antifungal protein PAF severely affects the integrity of the plasma membrane of *Aspergillus nidulans* and induces an apoptosis-like phenotype.. Antimicrob Agents Chemother.

[55] Qi GF, Zhu FY, Du P, Yang XF, Qiu DW (2010). Lipopeptide induces apoptosis in fungal cells by a mitochondria-dependent pathway.. Peptides.

[56] Starkov AA, Fiskum G (2003). Regulation of brain mitochondrial H_2_O_2_ production by membrane potential and NAD(P)H redox state.. J Neurochem.

